# Acquiring hepatitis C in prison: the social organisation of injecting risk

**DOI:** 10.1186/s12954-015-0045-2

**Published:** 2015-04-24

**Authors:** Carla Treloar, Luke McCredie, Andrew R Lloyd, on behalf of the HITS-p investigators

**Affiliations:** Centre for Social Research in Health, UNSW Australia, Sydney, NSW Australia; Centre for Health Research in Criminal Justice, Sydney, Australia; Inflammation and Infection Research Centre, School of Medical Sciences, UNSW Australia, Sydney, Australia

**Keywords:** Prison, Hepatitis C, Injecting drug use, Incidence, Australia, Harm reduction, Qualitative, Needle exchange

## Abstract

**Aim:**

The potential for transmission of hepatitis C virus (HCV) in prison settings is well established and directly associated with sharing of injecting and tattooing equipment, as well as physical violence. This study is one of the first to examine the circumstances surrounding the acquisition of HCV in the prison setting via inmates’ own accounts.

**Method:**

This is a sub-study of a cohort of prison inmates in New South Wales, Australia. Cohort participants were inmates who had reported ever injecting drugs and who had a negative HCV serological test within 12 months prior to enrolment. Cohort participants were monitored every 3 to 6 months for HCV antibodies and viraemia and via behavioural risk practices questionnaire. Participants with a documented HCV seroconversion were eligible to participate in in-depth interviews with a research nurse known to them.

**Results:**

Participants included six inmates (four men, two women) with documented within-prison HCV seroconversion. Participants reported few changes to their injecting practices or circumstances that they attributed to HCV acquisition. Participants believed that they were sharing syringes with others who were HCV negative and trusted that others would have declared their HCV status if positive. Some participants described cleaning equipment with water, but not with disinfectant. In a departure from usual routine, one participant suggested that he may have acquired HCV as a result of using a syringe pre-loaded with drugs that was given to him in return for lending a syringe to another inmate. Participants described regret at acquiring HCV and noted a number of pre- and post-release plans that this diagnosis impacted upon.

**Conclusions:**

Acquiring hepatitis C was not a neutral experience of participants but generated significant emotional reactions for some. Decisions to share injecting equipment were influenced by participants’ assumptions of the HCV status of their injecting partners. The social organisation of injecting, in trusted networks, is a challenge for HCV prevention programs and requires additional research.

## Introduction

Prisons are key settings for transmission of hepatitis C virus (HCV) infection [1,2]. The illegality of injecting drug use and subsequent high rates of incarceration of people who inject drugs combined with the lack of access to sterile injecting equipment places inmates at high risk of HCV infection [3-5]. Cohort studies indicate significant rates of HCV transmission in the custodial setting—particularly among inmates who inject drugs [1,2,6]. In 2010, prevalence of hepatitis C was 22% among Australian prison entrants and 51% among those with a history of injecting drug use [7].

Besides the high prevalence of HCV infection among those coming into prison, injecting drug use has been described as “normative” within prisons [8] with evidence of inmates starting to share injecting equipment within prison [9]. Furthermore, the lack of access to sterile injecting equipment means that such equipment becomes commodified and circulates for long periods as broken equipment is harvested to build or repair others [10,11]. In these situations, it appears impossible not to share injecting equipment and, hence, produce elevated risks of HCV transmission [12].

These findings argue for better understanding of the situations in which HCV transmission occurs in prisons to guide possible responses. This paper aims to address this issue via a qualitative study of continuously incarcerated inmates with serologically documented incident HCV infection. This paper sought to provide a contextualised understanding of the individual, social and environmental circumstances in which HCV was acquired by prison inmates. This project used qualitative research methods to allow participants to fully discuss and explore the practices and settings in which they perceived HCV transmission to have occurred.

## Method

Participants enrolled in the HITS-p cohort were eligible for this qualitative study. The HITS-p cohort is a prospective cohort of hepatitis C uninfected inmates who report injecting drug use [1,13]. Appropriate human research ethics committees (Corrective Services NSW, Justice Health and Forensic Mental Health Network) provided approval for the HITS-p cohort and for this project. At enrolment to the cohort, all participants were screened for HCV antibodies and HCV viraemia and then monitored every 3 to 6 months via blood test and interviewer-administered questionnaire to record behavioural risk practices (particularly, injecting drug use, tattoos and fights). In our recent report of HCV incidence in the cohort [1], the rate among continuously incarcerated inmates was 10.3 per 100 person years. This analysis, however, did not provide an opportunity to explore the HCV infection experience from the participant’s perspective.

Participants with a documented incident HCV infection (by antibody status) and who remained in prison were eligible to be invited to participate in in-depth interviews with a research nurse (LM) known to them via the cohort project. After written informed consent, qualitative interviews were conducted within 1 to 6 months of diagnosis (that is, not immediately upon diagnosis but within a period of time to facilitate recall of the events/settings surrounding acquisition of hepatitis C). At the conclusion of each interview, participants were offered written information about hepatitis C, an opportunity to discuss any further issues with the research nurse, and information about access to the Prison Hep C Infoline. Participants received AUD$10 for their participation in the interview through the approved prison inmate banking system to compensate for their time and effort in completing the research interview.

The interview schedule included topics such as risk (what risks are perceived by inmates; what risks can be compromised or negotiated and what cannot); participant’s knowledge of hepatitis C, hepatitis C information sources and perception of susceptibility to hepatitis C; and injecting drug use, tattooing and violence (including details of how, where, when, and with whom these activities occur; decisions/influences on safety and practice). Participants were asked to discuss how they believe they came to be infected with hepatitis C; had injecting practice changed since diagnosis; and the importance of knowing how HCV was acquired. Demographic information was collected from all participants.

Interviews were audio-taped and transcribed verbatim. Transcripts were checked for accuracy against recordings, de-identified and cleaned. The data was then read closely, and a number of themes were identified as relevant to the research questions. The research team then collaborated on the construction of a “coding frame”—a set of organising, interpretive themes to aid analysis. The coding frame was then used to organise interview data within NVivo 9. Analysis was informed by both a deductive and inductive approach [14]. That is, each case of incident HCV infection was reviewed to examine the specific circumstances in which transmission may have occurred as these data, from the participants’ perspective, have not been previously reported. To extend the presentation of these cases, two specific risk factors for HCV transmission (cleaning of equipment and injecting networks) were examined using an inductive thematic analysis approach. A final theme emerging from the data, responses to HCV diagnosis, is also presented. Each aspect of the thematic analysis, that is, the interpretations and meanings drawn from the interview data, was critically examined and summarised (along with supporting quotes). Quotes are presented by participant number and with details of their gender, age, frequency of injecting and HCV risk behaviours as reported on the behavioural survey preceding HCV acquisition.

## Results

Six participants (four males, two females) had documented incident HCV infection while continuously imprisoned, were available to interview and were asymptomatic. Two further participants also had documented incident infection, but one participant attributed this to injecting outside of prison and another terminated his interview before questions about HCV acquisition could be posed. Of the remaining six participants, two participants reported only injecting drug use as risk for HCV exposure in their responses to the behavioural risk practice questionnaire in the period corresponding to their HCV seroconversion; three participants reported injecting drug use and other possible exposure to blood (via tattooing, piercing, violence or haircuts) in the period around their acquisition of HCV. Data on sex between men was available for three of the four male participants in the time period of HCV acquisition; none of these male participants reported sex with men. One participant reported being on opiate substitution treatment around the time of seroconversion. The six participants were located in six different prisons at the time of the interview. Participants ranged in age from 22 to 31 years. Three participants identified as Anglo-Australian, two as Indigenous Australians and one as Polynesian. Five participants reported 10 years or less of formal schooling and one participant reported technical studies post-schooling. Participants’ reports of their current custodial sentence ranged from 14 months to 22.5 years.

### Circumstances of HCV acquisition

Participants’ accounts of the circumstances in which they believed that they acquired HCV are presented as unique cases. A theme across these accounts is the “routine” nature of HCV seroconversion (that is, participants could not identify changes in practice that could have resulted in increased HCV risk). However, alongside commentary about the “routine” nature of the suspected HCV seoconversion circumstances are instances in which participants made decisions to inject outside of routine (particularly with different injecting partners) because the opportunity to use drugs arose. Whatever the circumstances of the narrated events (routine or not), was that each participant placed trust in their injecting partners to provide protection against HCV (by declaring their positive status or cleaning equipment). Indeed, awareness of and attempts to clean equipment was a feature of each case and will be explored in detail below.

All but one participant described sharing of injecting equipment as the likely means of HCV transmission. One participant (#22, female, 23 years, daily injecting, tattoo, piercing), who reported injecting and other risks for HCV exposure, indicated that she did not know how she acquired HCV and similar to most of the remaining participants could find no change in routine that would explain exposure to HCV, including related to injecting drug use.

Participant #11 believed that he acquired HCV when he shared a needle/syringe with his cell mate who had previously disclosed his positive HCV status. This situation was different from typical injecting events in that the participant noted that he would usually inject with his cell mate and two other inmates. The participant and his cell mate injected together on this occasion as there were insufficient drugs to share with two other people. This participant held a strong belief that he would not acquire HCV.

I took the chance, yeah … I, I just thought I was, I don’t know, I had a gene in me body that’d beat it, you know. I never thought I’d catch it. Doesn’t matter how many, if I belted up bloody five units of blood, I wouldn’t catch it. And now I got it. But I never, I never thought I’d get it. (#11, male, 27 years, injecting more than once per day).

Participant #13 reported only injecting drugs within prison (not in the community) and that he had shared injecting equipment with the same person throughout the period of incarceration leading up to the incident infection. This participant could not describe how he was likely to have acquired hepatitis C at this time: “No, it was virtually the same as I do it any other time” (#13, male, 22 years, injecting daily).

Participant #18 indicated that he had acquired HCV “through shootin’ up. There’s not much else to say” and indicated that he assumed his regular and trusted injecting partner did not have hepatitis C. However, this participant also indicated that he may have used drugs with other people revealing the pragmatic decisions made to share equipment outside of regular networks to facilitate access to drugs.

I don’t think I’d used with anyone else. … But maybe I had. Maybe there was one or two times that I used with someone else. … [Interviewer: Why would you use with others?] Well probably ‘cause we didn’t have [drugs] and the only way for me to get was to use with them. (#18, male 27 years, injecting more than once per day, haircut where skin was cut)’.

Participant #21 described two possible scenarios in which HCV transmission could have occurred. One involved routine cleaning of equipment and the other a gift of a pre-mixed syringe after this participant had lent his syringe to another inmate. This account again raises the need to trust others in the situation of limited access to both equipment and drugs:

I knew the bloke who let me use the fit. I sort of, I cleaned it but you still, you still can’t know. The second time I lent my fit [needle/syringe] to a mate. He went and used it, brought it, brought it back and brought me back a shot. … it was already mixed up. And I said, “Did you clean it?” and he said, “Yeah”. And I was, I trusted the bloke. (#21, male 31 years, injecting monthly or more often, tattoo, fight where blood was present).

Participant #23 indicated that she preferred to smoke cannabis in gaol rather than inject drugs. She did, however, accept the offer of an injection because she could not access cannabis at that time and the two inmates involved in this instance declared that they were HCV negative. As an irregular injector, this participant also did not have her own equipment to use but relied on others to access this.

I prefer to smoke cones [of cannabis]. It’s just like that time when I did [inject] it was like no cones going around … Well there was just powders … I would prefer to buy a deal of pot than inject … [I got HCV through] sharing equipment. Sharing a needle. [Interviewer: So what, what happened? Like why did you share the needle?]’ Cause they swore that they didn’t have hepatitis C and I wanted to have a shot of Bupe [buprenorphine]. But I could have smoked it too. … So I had options. They just, it’s better the other way. … If I had that opportunity to be told [that people I would be injecting drugs with had HCV], I would have smoked it. I wouldn’t put the needle in my arm. (#23, female, 24 years, no injecting or other HCV risk behaviours reported).

### Cleaning of injecting equipment

Without access to sterile injecting equipment in prison, health promotion materials provide advice about cleaning equipment with water and a quaternary disinfectant provided in the NSW prisons, and accessible for general cleaning purposes. Common across all participants’ accounts was knowledge that cleaning of equipment was recommended to reduce the risk of HCV transmission and all but one participant attempted to put these protocols into practice. Among those who attempted cleaning, external factors were described as influencing the success of these attempts. Being able to access disinfectant and having the time to clean equipment without coming to the attention of correctional staff were significant barriers to following recommended cleaning protocols.

Just being able to have the, the time and the, just kind of like cleaning stuff around. … Well cleaning the, the syringe out properly and having enough time to do it without an officer or, you know, someone else trying to come, come in. (#21, male, 31 years, injecting monthly or more often, tattoo, fight where blood was present).

Despite this level of awareness and practice of cleaning, some participants revealed ambivalence about the advice and practice. Among this sample, there was resignation about the effectiveness of cleaning that was reinforced by participants’ subsequent HCV seroconversion: that cleaning equipment cannot guarantee safety or prevent HCV.

Clean it with water then [disinfectant], then water again. .. Well it’s not really safe. It’s just cleaning it (#22, female, 23 years, injecting daily, tattoo, piercing).

Well I’ve shared needles. [Disinfected] it. What it says to do, you know. Like three water, three [disinfectants], three water, and I still caught hep C, you know. (#23, female, 24 years, no injecting or other HCV risk behaviours reported).

Participant #11 had a unique attitude to cleaning in that he equated cleaning with “weakness”. Although this attitude was unique, it was also influenced by the need to rush to avoid detection and lack of access to bleach products (as opposed to the disinfectant provided).

Usin’ a needle and, you know, it’s not clean or you got no bleach no more. What we got runnin’ water and half the time … there was a, always a saying when I first come here, “water’s for weak cunts”. … Who needs cleaning? Just get, let’s get it over and done with, you know, ‘cause we’ve got two minutes before the screws [corrections officers] walk through the gate, you know. Let’s get this done quick. … And I’ve got [hepatitis C] because of that stupid, bloody little ruling I made up, (#11, male, 27 years, injecting more than once per day).

### Sharing injecting equipment

In situations of acute limitations on access to sterile equipment, inmates who inject must make decisions about who they will share equipment with. Participants’ accounts were heavily invested with notions and judgements of trust.

Participants relied on existing trusted relationships (some from prior to imprisonment) to make decisions about with whom to share injecting equipment. Assessments of trustworthiness were based on perceptions of hygiene and cleanliness (for example, how frequently inmates showered), the ways in which inmates carried themselves in the prison (that is, avoiding those who drew too much attention because of their behaviour) and those who looked drug affected (which may also draw unwanted attention from correctional staff).

not dickheads … Just carry on like fuckwits, like just being dumb cunts. Putting themselves out there. … And like I don’t mean to be like rude or anything but I normally go by looks. If you’re decent looking then, yeah …But, if you look like a junkie, I’m not gonna let you do a thing (#22, female, 23 years, injecting daily, tattoo, piercing).

In relation to HCV, participants relied on other inmates to disclose their HCV status and used this knowledge to form injecting networks and make decisions about managing HCV risk. By acquiring HCV, participants had become aware either of the mistaken trust they had in their own assumptions or the declarations of others.

I was shootin’ up with a bloke who I assumed didn’t have it. For no other reason than I was an idiot … ‘Cause I trusted him. … ‘Cause I’d known him a long time before I started shootin’. (#18, male 27 years, injecting more than once per day, haircut where blood was present).

a lot of people specifically lie to your face just to have a shot. You know what I mean? [Interviewer: About what, what do they lie to you about?] That they don’t have anything. … The two people I shared with swore to me on their kids that they didn’t have hep C. Well why the fuck do I have it? So that’s the way I look at it, you know. And that’s how low they will stoop, to swear on their own kids, to say that they don’t have [hepatitis C]. Because, if they told me, I was happy just to smoke it. (#23, female, 24 years, no injecting or other HCV risk behaviours reported).

### Responses to HCV diagnosis

The diagnosis of HCV was an emotionally charged experience for some participants with reports of anger and blame (at others who transmitted HCV infection) and regret (particularly in relation to cleaning of equipment). These reactions were also fed by concerns about HCV interfering with pre- and post-release plans, the association of HCV status with a “junkie” identity and the fear that participants could also have acquired HIV (perceived as a more serious and life-threatening infection than HCV) were also evident.

And then it shattered me. … It’s a blood virus that I don’t want, you know. Junk-bags got it and I’ve got it. … And now I joined ‘em. You know what I mean? I’m, I’ve got hepatitis. That’s the way I look at it: I’m a junkie. … I shouldn’t have had it. I shouldn’t have done what I was doin’, you know. I kick meself every day. (#11, male, 27 years, injecting more than once per day).

Participants’ reactions to diagnosis also generated varying responses to those who were perceived to be involved in HCV transmission and varying impacts on subsequent injecting practice. One of the six participants reported acting on her anger at acquiring HCV and confronted the person they believed was involved in HCV transmission. The reactions of these participants were grounded more in feelings of self-regret in relation to cleaning.

I don’t hate him ‘cause he gave it to me. I blame myself more than anyone and so it’s my fault that I got hep c - not his fault. I should have been more stringent on the cleaning process (#18, male 27 years, injecting more than once per day, haircut where skin was cut).

The impact of HCV diagnosis on injecting was varied from no change, to changes in drug use practice, and for some, a ceasing of injecting drug use. Two participants reported a change in practice following HCV infection. One participant reported restricting his injecting network to only one other person who had previously revealed a HCV positive status. The other participant reported a change in cleaning practices to avoid further HCV infections.

a bit more vigilant in the cleaning process. … Before maybe I might have lapsed, you know. But now it’s vigilant, every, you’ve gotta make sure you have clean every time … ‘cause I don’t wanna get more strands or genotypes (#18, male 27 years, injecting more than once per day, haircut where skin was cut).

Two participants reported ceasing injecting drug use and one of these was as a result of the “wake-up call” that the HCV diagnosis provided. The other participant had ceased injecting before being notified of their HCV diagnosis in an effort to “get cleaned up” (#13, male, 22 years, injecting daily) prior to release from prison.

Yeah, it was just a wake-up call, really. You know what I mean? Like to stop … it was a couple of months from when I used and then I flushed the equipment, you know, the fits. … to me it was like a wake-up call. I had to (#21, male 31 years, injecting monthly or more often, tattoo, fight where blood was present).

## Discussion

It is unsurprising that these participants described sharing of injecting equipment as leading to HCV transmission as this is a well-established risk factor for HCV infection, compounded by lack of prison programs for distribution of sterile equipment in most countries of the world [15]. Indeed, the factors that participants noted such as the importance of social networks, the difficulties in cleaning equipment, the commodification of equipment (resulting in the gift of a pre-mixed syringe) and the attitude of some prisoners towards their health have been raised in previous literature [8,10,16-18].

Inmates who inject drugs typically do so within prison at lower rates than in the community [13,19], and there is evidence that some inmates initiate injecting in prison [20]. Decisions to inject in prison, may be related to a number of factors including untreated dependence on illicit drugs, boredom and social bonding [17,21]. In the absence of prison-based needle and syringe programs, the harm reduction strategy available to inmates who inject is cleaning equipment with disinfectant via a recommended protocol of three rinses with water followed by three rinses with disinfectant, and three further rinses with water. These participants were all aware of these recommendations, but some reported that they did not put these into practice. Restrictions on the time available to clean equipment in the prison environment without coming to the attention of the correctional staff were noted. Such situational limitations on cleaning have been previously shown to modify the protective effect of equipment cleaning [22]. However, there was also a sense of fatalism for some participants noting that cleaning was “not safe” and that HCV transmission had occurred despite following cleaning recommendations. In addition, a recent meta-analysis suggested bleaching had no effect on HCV transmission [23] and epidemiological analysis of associations with incident HCV infection in the HITS-p cohort, those who reported “always” using disinfectant had no reduction in HCV incidence [1].

The importance of the organisation of injecting drug use in trusted social networks is another difficult area for prison health agencies to address [24]. Previous writers have examined the fears held by HIV positive inmates of being excluded from injecting opportunities, and social networks, if they disclose their status [10]. These findings suggest that similar social influences are at play in decisions about disclosure of HCV [25,26], despite the much higher prevalence of this infection than HIV. However, knowing (or assuming) others’ HCV status was one key element of decisions about injecting partners and negotiating HCV risk, across a range of prisons with differing accommodation and other organisational arrangements. As prisoners are moved between prisons, these networks will form and re-form. How best to address the social organisation of drug use requires further research and the input of prisoners in any resultant responses to examine both the risk and the positive outcomes of social relationships within prison [16].

This is the first study to examine the accounts of continuously incarcerated inmates who have acquired HCV within prison. The findings of this small study of six prison inmates cannot be generalised to the population of inmates at risk of HCV. However, this study was carefully designed to provide participants with the opportunity to undertake an interview with a trusted research nurse, who was not an employee of the corrections authorities, and who was known to participants via their regular participation in the HITS-p cohort. Also, data was collected within close temporal proximity to acute infection, largely averting issues of recall and other biases that may occur within studies of ex-inmates. Although it is well known that prison inmates can acquire multiple HCV infections [27], this account focused on their first confirmed HCV infection to avoid discussion of multiple possible events of risk over periods of time. These findings also highlight the usefulness of qualitative data in making sense of epidemiological and behavioural surveillance data. When responding to the behaviour survey, participant #23 reported no injecting or other HCV risk behaviours at the time of HCV acquisition. However, her narrative account clearly demonstrates that she did inject, although this was an unusual occurrence which could have accounted for the response she gave to the survey question. It is also interesting to note that while three of the six participants reported other exposures to blood (via tattooing, piercing, violence and haircuts), their accounts of HCV acquisition focused exclusively on injecting drug use.

It is recognised that no single intervention is effective for the prevention of HCV [23] and combined approaches are recommended for both prevention of HIV [28,29] and HCV among PWID [30,31]. Participants’ accounts focused on reuse of equipment and inadequate use of disinfectant as determining factors of HCV acquisition. Another strategy shown to effect HCV risk is the provision of opiate substitution treatment (OST). In NSW prisons, methadone and buprenorphine were provided to opioid-dependent inmates by a health service that operates independently of corrections authorities [32,33]. One participant was receiving OST despite four reporting daily or more frequent injection. There is long-standing evidence of the benefits of OST on numerous health and well-being measures [34] and more recent evidence has shown the impact of OST on reduction of reincarceration rates [35]. OST of adequate dose and duration has the potential to impact the “pool” of people injecting within the prison environment by decreasing frequency of injecting among those on OST [36,37] and should be considered as part of a package of HCV prevention strategies.

Addressing the risk of HCV acquisition among prison inmates is complex when distribution of sterile equipment is prohibited, equipment cleaning procedures are difficult to enact (or are ineffective) and prisoners may not have access to OST. This study adds to the evidence that inmates are required to make difficult choices about risk, and risk reduction. What should also be noted is that without access to services equivalent to community standards (that is, access to harm reduction measures), HCV infections continue to occur among the most vulnerable, marginalised and excluded members of our society compounding the ill-health and social position of these individuals as well as generating significant societal and health care costs related to the management of these infections.

## References

[1] Luciani F, Bretaña N, Teutsch S, Amin J, Topp L, Dore G (2014). A prospective study of hepatitis C incidence in Australian prisoners. Addiction.

[2] Larney S, Kopinski H, Beckwith C, Zaller N, Des Jarlais D, Hagan H (2013). Incidence and prevalence of hepatitis C in prisons and other closed settings: results of a systematic review and meta-analysis. Hepatology.

[3] Vescio M, Longo B, Babudieri S, Starnini G, Carbonara S, Rezza G (2008). Correlates of hepatitis C virus seropositivity in prison inmates: a meta-analysis. J Epidemiol Community Health.

[4] Awofeso N (2010). Prisons as social determinants of hepatitis C virus and tuberculosis infections. Public Health Rep.

[5] Post J, Arain A, Lloyd A (2013). Enhancing assessment and treatment of hepatitis C in the custodial setting. Clin Infect Dis.

[6] Champion J, Taylor A, Hutchinson S, Cameron S, McMenamin J, Mitchell A (2004). Incidence of hepatitis C virus infection and associated risk factors among Scottish prison inmates: a cohort study. Am J Epidemiol.

[7] Butler T, Lim D, Callander D (2011). National prison entrants’ bloodborne virus and risk behaviour survey report 2004, 2007, and 2010: prevalence of HIV, HBV, HCV, and risk behaviours among Australian prison entrants: national report.

[8] Bonnycastle KD (2011). Injecting risk into prison sentences: a quantitative analysis of a prisoner-driven survey to measure HCV/HIV seroprevalence, risk practices and viral testing at one Canadian male federal prison. Prison J.

[9] Ford P, Perason M, Sankar-Mistry P, Stvenson T, Bell D, Austin J (2000). HIV, hepatitis C and risk behavior in a Canadian medium-security federal penitentiary [for men]. Queens J Med.

[10] Small W, Kain S, Laliberte N, Schechter M, O’Shaughnessy MV, Spittal P (2005). Incarceration, addiction and harm reduction: inmates experience injecting drugs in prison. Subst Use Misuse.

[11] Dyer J, Tolliday L (2009). Hepatitis C education and support in Australian prisons: preliminary findings of a nationwide survey. Health Promot J Austr.

[12] Kinner S, Jenkinson R, Gouillou M, Milloy M (2012). High-risk drug -use practices among a large sample of Australian prisoners. Drug Alcohol Depend.

[13] Dolan K, Teutsch S, Scheuer N (2010). Levy M.

[14] Braun V, Clarke V (2006). Using thematic analysis in psychology. Qual Res Psychol.

[15] Dolan K, Rutter S, Wodak A (2003). Prison-based syringe exchange programmes: a review of international research and development. Addiction.

[16] de Viggiani N (2007). Unhealthy prisons: exploring structural determinants of prison health. Sociol Health Illn.

[17] Hughes R, Huby M (2000). Life in prison: perspectives of drug injectors. Deviant Behav.

[18] Turnbull P, Power R, Stimson G (1996). “Just using old works”: injecting risk behaviour in prison. Drug Alcohol Rev.

[19] Butler T, Kariminia A, Levy M, Kaldor J (2004). Prisoners are at risk for hepatitis C transmission. Eur J Epidemiol.

[20] Boys A, Farrell M, Bebbington P, Brugha T, Coid J, Jenkins R (2002). Drug use and initiation in prison: results from a national prison survey in England and Wales. Addiction.

[21] Jürgens R, Ball A, Verster A (2009). Interventions to reduce HIV transmission related to injecting drug use in prison. Lancet Infect Dis.

[22] Kapadia F, Vlahov D, Des Jarlais D, Strathdee S, Ouellet L, Kerndt P (2002). Does bleach disinfection of syringes protect against hepatitis C infection among young adult injection drug users?. Epidemiology.

[23] Hagan H, Pouget E, Des JD (2011). A systematic review and meta-analysis of interventions to prevent hepatitis C virus infection in people who inject drugs. J Infect Dis.

[24] Bonnycastle KD (2011). The social organisation of penal tattooing in two Canadian federal male prisons: locating sites of risk for empirically-based health care interventions. Howard J Crim Justice.

[25] Khaw F, Stobbart L, Murtagh M (2007). ‘I just keep thinking I haven’t got it because I’m not yellow’: a qualitative study of the factors that influence the uptake of Hepatitis C testing by prisoners. BMC Public Health.

[26] Yap L, Carruthers S, Thompson S, Cheng W, Jones J, Simpson P (2014). A descriptive model of patient readiness, motivators, and hepatitis C treatment uptake among Australian prisoners. PLoS One.

[27] Pham S, Bull R, Bennett J, Rawlinson W, Dore G, Lloyd A (2010). Frequent multiple hepatitis C virus infections among injection drug users in a prison setting. Hepatology.

[28] Degenhardt L, Mathers B, Vickerman P, Rhodes T, Latkin C, Hickman M (2010). Prevention of HIV infection for people who inject drugs: why individual, structural and combination approaches are needed. Lancet.

[29] UNODC (2013). HIV prevention, treatment and care in prisons and other closed settings: a comprehensive package of interventions.

[30] Van Den Berg C, Smit C, Van Brussel G, Coutinho R, Prins M (2007). Amsterdam Cohort. Full participation in harm reduction programmes is associated with decreased risk for human immunodeficiency virus and hepatitis C virus: evidence from the Amsterdam Cohort Studies among drug users. Addiction.

[31] Turner K, Hutchinson S, Vickerman P, Hope V, Craine N, Palmateer N (2011). The impact of needle and syringe provision and opiate substitution therapy on the incidence of hepatitis C virus in injecting drug users: pooling of UK evidence. Addiction.

[32] Levy M, Treloar C, McDonald R, Booker N (2007). Prisons and bloodborne viruses: old challenges, new solutions. Med J Aust.

[33] Levy M, Treloar C (2012). Harm minimisation in Australian prisons—health protection still depends on where you serve your time [letter]. Med J Aust.

[34] Hall W, Ward J, Mattick R, Ward J, Mattick RP, Hall W (1998). The effectiveness of methadone maintenance treatment 1: Heroin use and crime. Methadone maintenance treatment and other opioid replacement therapies.

[35] Larney S, Toson B, Burns L, Dolan K (2012). Effect of prison-based opioid substitution treatment and post-release retention in treatment on risk of re-incarceration. Addiction.

[36] Warren E, Viney R, Shearer J, Shanahan M, Wodak A, Dolan K (2006). Value for money in drug treatment: economic evaluation of prison methadone. Drug Alcohol Depend.

[37] Dolan K, Wodak A, Hall W (1998). Methadone maintenance treatment reduces heroin injection in New South Wales prisons. Drug Alcohol Rev.

